# Endovascular treatment of traumatic subclavian arteriovenous fistula: case report

**DOI:** 10.1590/1677-5449.010317

**Published:** 2018

**Authors:** José Júlio Bechir Maués, Heather Lynn Hauter

**Affiliations:** 1 Hospital Saúde da Mulher – HSM, Departamento de Cirurgia Vascular e Endovascular, Belém, PA, Brasil.

**Keywords:** arteriovenous fistula, vascular system injuries, wounds and injuries, endovascular procedures

## Abstract

A 47-year-old male police officer presented at an outpatients consulting room complaining of exertional dyspnea and swelling and pain in the right arm. He had suffered a perforating gunshot wound of the right infraclavicular region 7 months previously. A chest tomography showed considerable dilatation of the subclavian and cervical veins and veins of the right upper limb, with no clear point of arteriovenous communication. His symptoms exacerbated and he was admitted to hospital before the date scheduled for treatment. He underwent endovascular treatment with an 8x100 mm Fluency covered stent that was placed in the right subclavian artery using the through-and-through technique. Control angiography showed that the fistulous defect had been completely sealed. There was significant relief of the symptoms on the first day after the operation. Traumatic lesions of the subclavian artery are rare, but can be associated with high morbidity and mortality rates. Penetrating trauma is the main cause and arteriovenous fistulas should be ruled out when evaluating penetrating injuries in vascular territories.

## INTRODUCTION

 Arteriovenous fistulas are anomalous communications between arteries and veins and may be congenital or acquired. Acquired fistulas can be caused by penetrating or blunt traumas or by endovascular or surgical procedures. 1 ^,^ 2 A traumatic arteriovenous fistula was first described in 1757, and was caused by puncture of the basilic vein performed to bleed the patient. 2 Traumatic fistulas caused by firearm wounds are generally the result of low velocity projectiles. 2 Unidentified traumatic arteriovenous fistulas can cause heart failure, murmur, and thrill, and, when located in limbs, may manifest with edema and reduced perfusion. Here, we describe a case in which a firearm projectile wound caused formation of a fistula between the right subclavian vessels, with late clinical manifestations. 

## CASE DESCRIPTION

 The patient was a 47-year-old male police officer who sought care at a consulting office and had been the victim of a perforating firearm wound to the right infraclavicular region 7 months prior to presentation. At the time of wounding he had been treated conservatively. 

 The patient complained of exertional dyspnea and considerable edema and pain in the right arm. He had brought the results of a chest tomography conducted some weeks before which showed considerable dilatation of the right subclavian vein and the cervical veins of the right upper limb. 

 Physical examination revealed significant edema of the right upper limb, with pain on palpation and holosystolic murmur in the topography of the right pulmonary apex. Right radial, ulnar, and brachial pulses were all reduced in comparison with those of the contralateral limb. 

 Two weeks after this consultation, the patient presented at an emergency room with exacerbation of the dyspnea, symptomatic ventricular tachycardia, and frequent premature ventricular contractions and was admitted to the hospital. 

 Supplementary cardiac tests were then conducted. The echocardiogram showed dilatation of the left cardiac chambers and an ejection fraction of 63%. Myocardial scintigraphy showed signs of dilated cardiomyopathy. 

**Figure 1:**
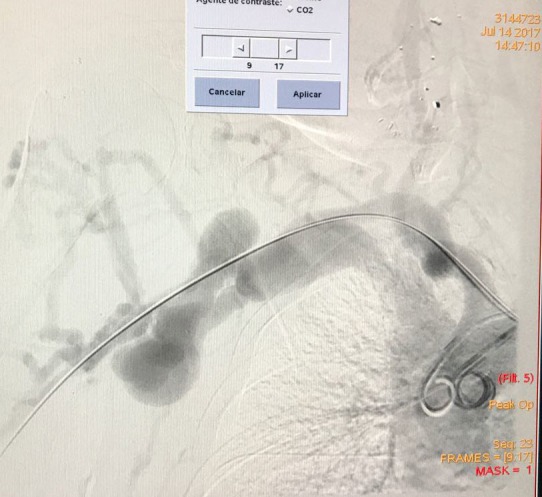
Arteriography prior to placement of the covered stent.

 After clinical and cardiac stabilization, the patient underwent arteriography of the right upper limb, which showed a large arteriovenous fistula between the right subclavian vessels and a pseudoaneurysm of the subclavian artery ( Figure 1 ). 

**Figure 2:**
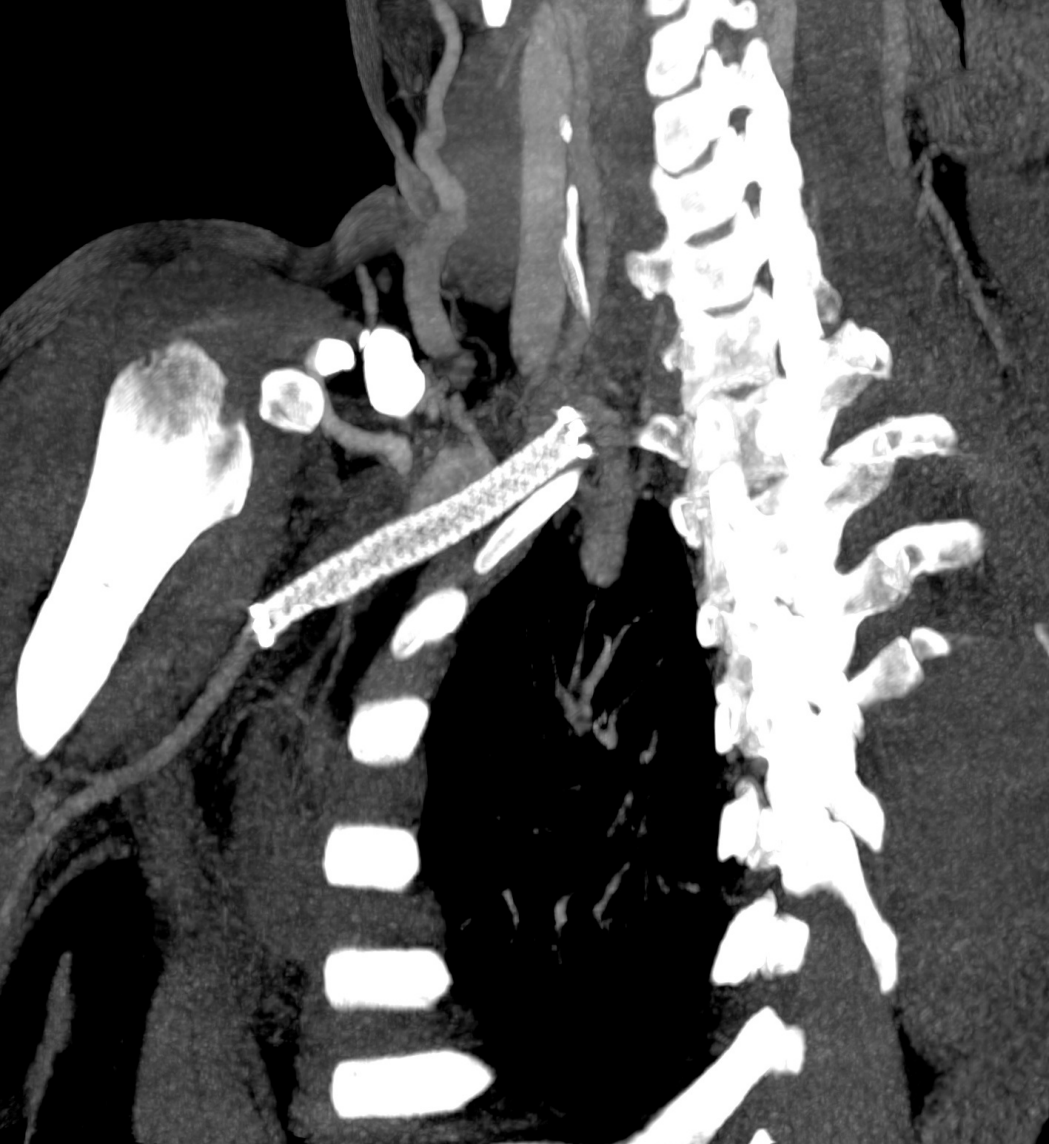
Placement of the covered stent.

 The treatment chosen was endovascular repair under local anesthesia with sedation. The technique employed was via puncture of the right common femoral artery with a 7F introducer and puncture of the right brachial artery with a 5F introducer. The subclavian artery was catheterized via the brachial access and the guidewire was snared and a through-and-through system constructed via the femoral access, due to difficulty in advancing the guidewire via the subclavian artery. The injury was repaired using a 8x100 mm Fluency covered stent (Bard) ( Figure 2 ). 

 After the procedure, the patient was transferred to the ward. He exhibited good postoperative recovery, with significant improvement of the pain in the right upper limb and reestablishment of symmetry of pulses with the contralateral limb. He was discharged from the hospital 2 days after the operation, on double platelet antiaggregation with acetylsalicylic acid and clopidogrel. 

 He was reassessed 15 days later in the consulting room. There was regression of the right upper limb edema, maintenance of the radial, ulnar, brachial pulses, and improvement of the dyspnea. 

**Figure 3:**
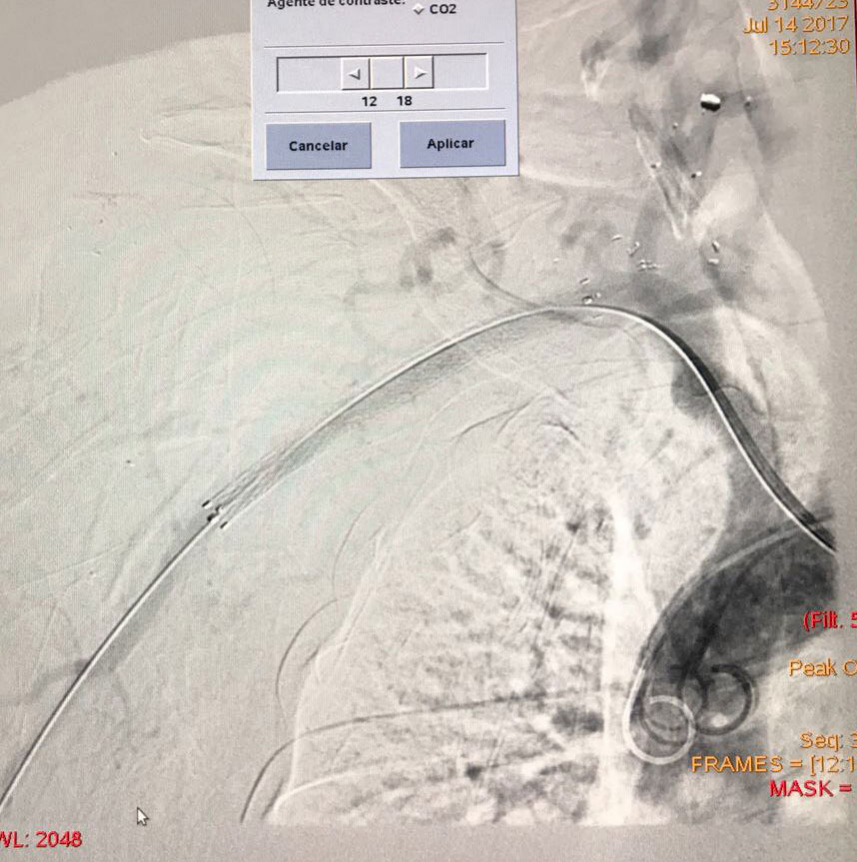
Postoperative angiotomography.

 A control angiotomography conducted 15 days after the follow-up visit (i.e., 30 days after the procedure) showed that the endoprosthesis was patent and there was no premature venous filling ( Figure 3 ). 

 The study was approved by the Research Ethics Committee at the Hospital Saúde da Mulher (HSM), Belém, PA, Brazil. 

## DISCUSSION

 Traumatic injuries of the subclavian arteries are uncommon, but are associated with elevated morbidity and mortality rates. 2 ^-^ 4 Penetrating traumas are the most common causes. 3 Incidence ranges from 2.3% to 3.9%. 4 ^-^ 6 In a review of 262 cases of traumatic fistulas, Rich et al. 6 found one case of fistula of subclavian vessels. 6

 Open surgery to treat traumatic lesions of the subclavian vessels can be a challenge because of their proximity to highly important neurovascular structures, in addition to presence of edema, hematoma, and anatomic changes caused by the trauma. 7 Postoperative mortality ranges from 5 to 40%, 3 ^,^ 4 ^,^ 8 and postoperative complications can reach 24%. 3

 Endovascular treatment is a safe and effective alternative to open surgery. 9 Morbidity and mortality are in the range of 5 to 10% among patients treated using endovascular methods. 3 ^,^ 5 Endovascular surgery is possible in about 50% of cases of traumatic subclavian artery injury. 10 ^-^ 12

 Endovascular treatment can be used for intimal injuries, dissection, fistulas, and pseudoaneurysms. The main contraindications are long injuries and absence of a proximal neck for anchoring, discrepancy between the proximal and distal diameters of vessels, impossibility of catheterization of the target vessel, injuries requiring surgical exploration, hematoma with compressive symptoms, and infected injuries. 3 ^,^ 7

 Short and medium-term results have revealed low rates of occlusion and intrastent stenosis. 7 ^,^ 8 ^,^ 13 Even in cases in which occlusion occurs, there is no impediment to revascularization when needed. There are reports of early failure of the covered stent in 5% of cases, which is comparable to results from literature reviews of open repairs of subclavian injuries. 14 ^,^ 15

 In the case described here, endovascular treatment was advantageous because of the site of the injury, significant venous dilatation with collateral vessels in both the supraclavicular and infraclavicular regions, the better postoperative recovery results, and lower blood loss. 

 Durability of endovascular prostheses has not yet been entirely established and there are no reports of long-term follow-up of such cases. For this reason, there is no consensus on routine use of endovascular treatment for subclavian artery injuries. There is therefore a need for a review of cases and of long-term follow-up. 

 This report demonstrates the importance of ruling out vascular injuries in cases where penetrating lesions cross the paths of vessels, even when they seem to be of little importance during the initial evaluation and treatment. 
