# Supplementary material for: Endovascular treatment of traumatic subclavian arteriovenous fistula: case report
Source: J Vasc Bras. 2018 Jul-Sep;17(3):248–51. doi: 10.1590/1677-5449.010317 (PMC6326127; doi:10.1590/1677-5449.010317)
Supplement: Translation

# Translation: Tratamento endovascular de fístula traumática de vasos subclávios: relato de caso

**Authors:** José Júlio Bechir Maués, Heather Lynn Hauter

**Affiliations:** 1 Hospital Saúde da Mulher – HSM, Departamento de Cirurgia Vascular e Endovascular, Belém, PA, Brasil.

**Keywords:** fístula arteriovenosa, lesões do sistema vascular, ferimentos e lesões, procedimentos endovasculares

## Abstract

Policial masculino de 47 anos foi atendido em consultório com queixa de dispneia aos esforços, edema e dor importantes em braço direito. Relatou ferimento por arma de fogo infraclavicular direito 7 meses antes. Tomografia de tórax mostrou grande dilatação de veia subclávia, veias cervicais e de membro superior direito sem identificação da comunicação arteriovenosa. O paciente foi internado antes da data prevista para tratamento por piora clínica e foi submetido a implante de stent revestido Fluency 8x100 mm em artéria subclávia direita por técnica do varal. Angiografia de controle mostrou artéria subclávia pérvia e fechamento da fístula. Houve melhora dos sintomas em braço direito no primeiro dia após o procedimento. As lesões traumáticas da artéria subclávia são incomuns, porém podem evoluir com alto índice de morbimortalidade O trauma penetrante é o principal agente etiológico, e fístulas arteriovenosas devem ser pesquisadas durante o atendimento do paciente com lesões penetrantes em trajeto vascular.

## INTRODUÇÃO

 As fístulas arteriovenosas são comunicações anômalas entre artérias e veias que podem ser congênitas ou adquiridas. As últimas têm como causas traumas penetrantes ou contusos, ou procedimentos endovasculares ou cirúrgicos 1 ^,^ 2 . A primeira fístula arteriovenosa traumática, decorrente de sangria por punção de veia basílica, foi descrita em 1757 2 . As fístulas traumáticas por ferimento por arma de fogo geralmente são ocasionadas por projétil de baixa velocidade 2 . As fístulas arteriovenosas traumáticas não identificadas podem evoluir para quadros de insuficiência cardíaca, sopro, frêmitos e, quando localizadas nos membros, podem se manifestar com edema e diminuição da perfusão. Relatamos um caso de ferimento por projétil por arma de fogo que resultou na formação de fístula entre os vasos subclávios à direita com manifestação clínica tardia. 

## DESCRIÇÃO DO CASO

 Trata-se de paciente masculino, policial, 47 anos, que procurou atendimento em consultório e havia sido vítima de ferimento transfixante por arma de fogo em região infraclavicular direita 7 meses antes. Na ocasião, foi submetido a tratamento conservador. 

 O paciente queixava-se de dispneia aos esforços, edema e dor importantes em braço direito. Trazia tomografia de tórax realizada algumas semanas antes que mostrava dilatação importante da veia subclávia direita, assim como de veias cervicais e do membro superior direito. 

 Ao exame físico, o paciente apresentava edema importante de membro superior direito, com dor à palpação, e sopro holossistólico em topografia de ápice pulmonar direito. Os pulsos radial, ulnar e braquial estavam diminuídos em relação aos pulsos do membro contralateral. 

 Duas semanas após a consulta, o paciente procurou o pronto-socorro com piora da dispneia, taquicardia ventricular sintomática e extra-sístoles ventriculares frequentes, sendo necessária a internação hospitalar. 

 Exames cardiológicos complementares foram realizados durante a internação. O ecocardiograma evidenciou dilatação de câmaras cardíacas esquerdas e uma fração de ejeção de 63%. A cintilografia miocárdica mostrou sinais de miocardiopatia dilatada. 

 Após compensação clínica e cardiológica, o paciente foi submetido a uma arteriografia de membro superior direito, que mostrou uma grande fístula arteriovenosa em vasos subclávios direitos com pseudoaneurisma de artéria subclávia ( Figura 1 ). 

**Figura 1:** Arteriografia pré-implante de stent revestido.

 Optou-se pela correção endovascular da lesão com anestesia local e sedação. A técnica foi realizada por punção de artéria femoral comum direita com introdutor 7F e punção de artéria braquial direita com introdutor 5F. A artéria subclávia foi cateterizada pelo acesso braquial com confecção de varal e captura do fio-guia com snare por acesso femoral em virtude da dificuldade em fio-guia pela artéria subclávia. A lesão foi corrigida com um stent revestido Fluency 8x100 mm (Bard) ( Figura 2 ). 

**Figura 2:** Implante de stent revestido.

 Após o procedimento, o paciente foi encaminhado para a enfermaria. Apresentou boa evolução pós-operatória, com melhora significativa da dor no membro superior direito e restabelecimento da simetria dos pulsos com o membro contralateral. Recebeu alta hospitalar no segundo dia de pós-operatório, com dupla antiagregação plaquetária com ácido acetilsalicílico e clopidogrel. 

 Foi reavaliado após 15 dias no consultório, apresentando regressão do edema de membro superior direito, manutenção dos pulsos radial, ulnar e braquial e melhora da dispneia. 

 Angiotomografia de controle realizada 15 dias após o retorno (30 dias após o procedimento) evidenciou endoprótese pérvia e sem enchimento venoso precoce ( Figura 3 ). 

**Figura 3:** ANGIOGRAFIA pos operatoria.

 O estudo foi aprovado pelo Comitê de Ética em Pesquisa do Hospital Saúde da Mulher (HSM), Belém (PA). 

## DISCUSSÃO

 As lesões traumáticas das artérias subclávias são incomuns, porém estão associadas a elevados índices de morbidade e mortalidade 2 ^-^ 4 . O trauma penetrante é o principal responsável 3 . Sua incidência varia de 2,3% a 3,9% 4 ^-^ 6 . Em levantamento de 262 casos de fístulas traumáticas, Rich et al. 6 encontraram um caso de fístula de vasos subclávios. 

 A cirurgia aberta para o tratamento das lesões traumáticas dos vasos subclávios pode ser um desafio, devido à proximidade com estruturas neurovasculares nobres, além da presença de edema, hematoma e alterações anatômicas decorrentes do trauma 7 . A mortalidade pós-operatória varia entre 5 a 40% 3 ^,^ 4 ^,^ 8 , e as complicações pós-operatórias podem chegar a 24% 3 . 

 O tratamento endovascular apresenta-se como uma alternativa segura e eficaz à cirurgia aberta 9 . A morbimortalidade varia entre 5 e 10% nos pacientes abordados por método endovascular 3 ^,^ 5 . A abordagem endovascular é factível em cerca de 50% dos casos de lesão traumática da artéria subclávia 10 ^-^ 12 . 

 O tratamento endovascular pode ser realizado para lesões intimais, dissecção, fístulas, pseudoaneurismas. As principais contraindicações são as lesões longas e a ausência de colo proximal para fixação, discrepância entre os diâmetros proximal e distal dos vasos, impossibilidade de cateterização do vaso alvo, lesões que necessitam de exploração cirúrgica, hematoma com sintomas compressivos e lesões infectadas 3 ^,^ 7 . 

 Resultados a curto e médio prazo mostram taxas pequenas de oclusão e estenose intrastent 7 ^,^ 8 ^,^ 13 . Mesmo nos casos de oclusão, não há impedimento para revascularização, caso necessária. Houve relatos de falha precoce do stent revestido em 5% dos casos, comparável com revisão de literatura de falha em correções abertas de lesão de subclávia 14 ^,^ 15 . 

 No caso citado, o tratamento endovascular se mostrou vantajoso, devido à localização da lesão, dilatação venosa importante com presença de colaterais em região supra e infraclavicular, melhor recuperação pós-operatória e menor perda sanguínea. 

 A durabilidade das próteses endovasculares ainda não foi completamente estabelecida, e não há relatos de acompanhamento de longo prazo desses casos. Dessa forma, não há consenso quanto ao uso rotineiro do tratamento endovascular para as lesões de artéria subclávia, sendo necessário levantamento de casos e acompanhamento a longo prazo. 

 Este relato mostra a importância de excluir lesões na presença de ferimentos penetrantes em trajeto vascular, mesmo que ao primeiro atendimento pareçam lesões de menor importância.
